# Rare but Relevant? Assessing Variants in Dystonia‐Linked Genes in Parkinson's Disease

**DOI:** 10.1002/mds.70073

**Published:** 2025-10-11

**Authors:** Lara M. Lange, Zih‐Hua Fang, Laurel Screven, Ai Huey Tan, Roy N. Alcalay, Rim Amouri, Roberta Bovenzi, Matilda Fenn, Joshua L.I. Frost, Joseph Jankovic, Simona Jasaityte, Zane Jaunmuktane, Beomseok Jeon, Ignacio Juan Keller Sarmiento, Rejko Krüger, Gregor Kuhlenbäumer, Chin‐Hsien Lin, Lukas Pavelka, Maria Teresa Periñan, Samia Ben Sassi, Tommaso Schirinzi, Jung Hwan Shin, Joshua M. Shulman, Yi Wen Tay, Ryan Uitti, Tom Warner, Zbigniew K. Wszolek, Lesley Wu, Ruey‐Meei Wu, Kirsten E. Zeuner, Cornelis Blauwendraat, Andrew Singleton, Niccolò E. Mencacci, Huw R. Morris, Shen‐Yang Lim, Katja Lohmann, Christine Klein, the Global Parkinson's Genetics Program (GP2)

**Affiliations:** ^1^ Laboratory of Neurogenetics National Institute on Aging Bethesda Maryland USA; ^2^ Institute of Neurogenetics University of Luebeck Luebeck Germany; ^3^ German Center for Neurodegenerative Diseases (DZNE) Tübingen Germany; ^4^ Global Parkinson's Genetics Program (GP2) Chevy Chase Maryland USA; ^5^ Division of Neurology, Department of Medicine, Faculty of Medicine University of Malaya Kuala Lumpur Malaysia; ^6^ Neurological Institute, Tel Aviv Sourasky Medical Center Tel Aviv Israel; ^7^ Department of Neurology Columbia Irving Medical School New York New York USA; ^8^ National Institute Mongi Ben Hamida of Neurology Tunis Tunisia; ^9^ Neurology Unit, Department of Systems Medicine Tor Vergata University of Rome Rome Italy; ^10^ Department of Neurology Northwestern University Feinberg School of Medicine Chicago Illinois USA; ^11^ Department of Clinical and Movement Neurosciences UCL Queen Square Institute of Neurology London United Kingdom; ^12^ Department of Neurology, Parkinson's Disease Center and Movement Disorders Clinic Baylor College of Medicine Houston Texas USA; ^13^ Queen Square Brain Bank for Neurological Disorders UCL Queen Square Institute of Neurology London United Kingdom; ^14^ Division of Neuropathology, National Hospital for Neurology and Neurosurgery University College London NHS Foundation Trust London United Kingdom; ^15^ Department of Neurology Seoul National University Hospital Seoul South Korea; ^16^ Luxembourg Centre for Systems Biomedicine (LCSB) University of Luxembourg Esch‐sur‐Alzette Luxembourg; ^17^ Luxembourg Institute of Health (LIH), Strassen, Luxembourg and Centre Hospitalier de Luxembourg (CHL) Esch‐sur‐Alzette Luxembourg; ^18^ Department of Neurology University Hospital Schleswig‐Holstein (UKSH) Kiel Germany; ^19^ Department of Neurology National Taiwan University Hospital Taipei Taiwan; ^20^ Centre for Preventive Neurology, Wolfson Institute of Population Health Queen Mary University of London London United Kingdom; ^21^ Unidad de Trastornos del Movimiento, Servicio de Neurología y Neurofisiología Clínica, Instituto de Biomedicina de Sevilla, Hospital Universitario Virgen del Rocío/Consejo Superior de Investigaciones Científicas (CSIC)/Universidad de Sevilla Seville Spain; ^22^ Duncan Neurological Research Institute Texas Children's Hospital Houston Texas USA; ^23^ Mayo Clinic Jacksonville Florida USA; ^24^ Coalition for Aligning Science Chevy Chase Maryland USA; ^25^ UCL Movement Disorders Centre University College London London United Kingdom

**Keywords:** dystonia, monogenic, Parkinson's disease, *GCH1*, *VPS16*

## Abstract

**Background:**

Dystonia and Parkinson's disease (PD) exhibit clinical and genetic overlap, but the relevance of dystonia gene variants in PD remains unclear.

**Objective:**

The aim was to assess the frequency of dystonia‐linked pathogenic variants in PD.

**Methods:**

We screened sequencing data from 15,684 individuals (8272 PD, 3200 atypical parkinsonism, and 4212 unaffected) from the Global Parkinson's Genetics Program (GP2) and Accelerating Medicines Partnership‐Parkinson's Disease (AMP‐PD) for variants in genes linked to isolated dystonia, dystonia‐parkinsonism, and myoclonus‐dystonia.

**Results:**

Pathogenic variants were identified only in PD patients. Forty‐five PD individuals (0.54%) carried 26 distinct (likely) pathogenic variants in nine dystonia‐linked genes, most frequently in *GCH1*, followed by *VPS16*.

**Conclusion:**

Though rare, pathogenic variants in dystonia‐linked genes are present in clinically and pathologically diagnosed PD. Our results reinforce *GCH1* as a PD‐relevant gene with clinical implications, whereas variants identified in other genes are rare and of uncertain relation to the PD phenotype. © 2025 The Author(s). *Movement Disorders* published by Wiley Periodicals LLC on behalf of International Parkinson and Movement Disorder Society.

Dystonia is a clinically heterogeneous disorder. Its etiology includes nervous system pathologies, acquired causes, and genetic factors.^1^ To date, variants in over 400 genes have been linked to different forms of dystonia, though the majority are associated with more complex and broader neurological presentations.^2^, ^3^, ^4^, ^5^, ^6^, ^7^ The term “dystonia” is used not only to refer to a disease entity itself but also to describe a symptom as part of another neurological disorder. For example, dystonic symptoms are frequently reported in individuals with Parkinson's disease (PD), either related to dopaminergic treatment and motor fluctuations or as an initial disease manifestation, especially in early‐onset PD.^8^, ^9^ Dystonic symptoms are also commonly encountered in atypical parkinsonism, though the dystonic features typically differ from those in PD. Interestingly, previous screening studies of PD patients identified carriers of pathogenic variants in genes primarily linked to dystonia, most frequently *GCH1*.^10^, ^11^

Moreover, several neurogenetic conditions include features of both dystonia and parkinsonism, either individually or combined, that is, where dystonia and parkinsonism are equally prominent. Based on this phenotypic and genetic overlap between dystonia and parkinsonism, this study aimed to assess the frequency of pathogenic variants in dystonia genes in individuals with PD and atypical parkinsonism by leveraging large‐scale sequencing data from the Global Parkinson's Genetics Program (GP2, https://gp2.org/)^12^, ^13^ and the Accelerating Medicines Partnership‐Parkinson's Disease (AMP‐PD, https://www.amp-pd.org/).

## Patients and Methods

**FIG. 1:**
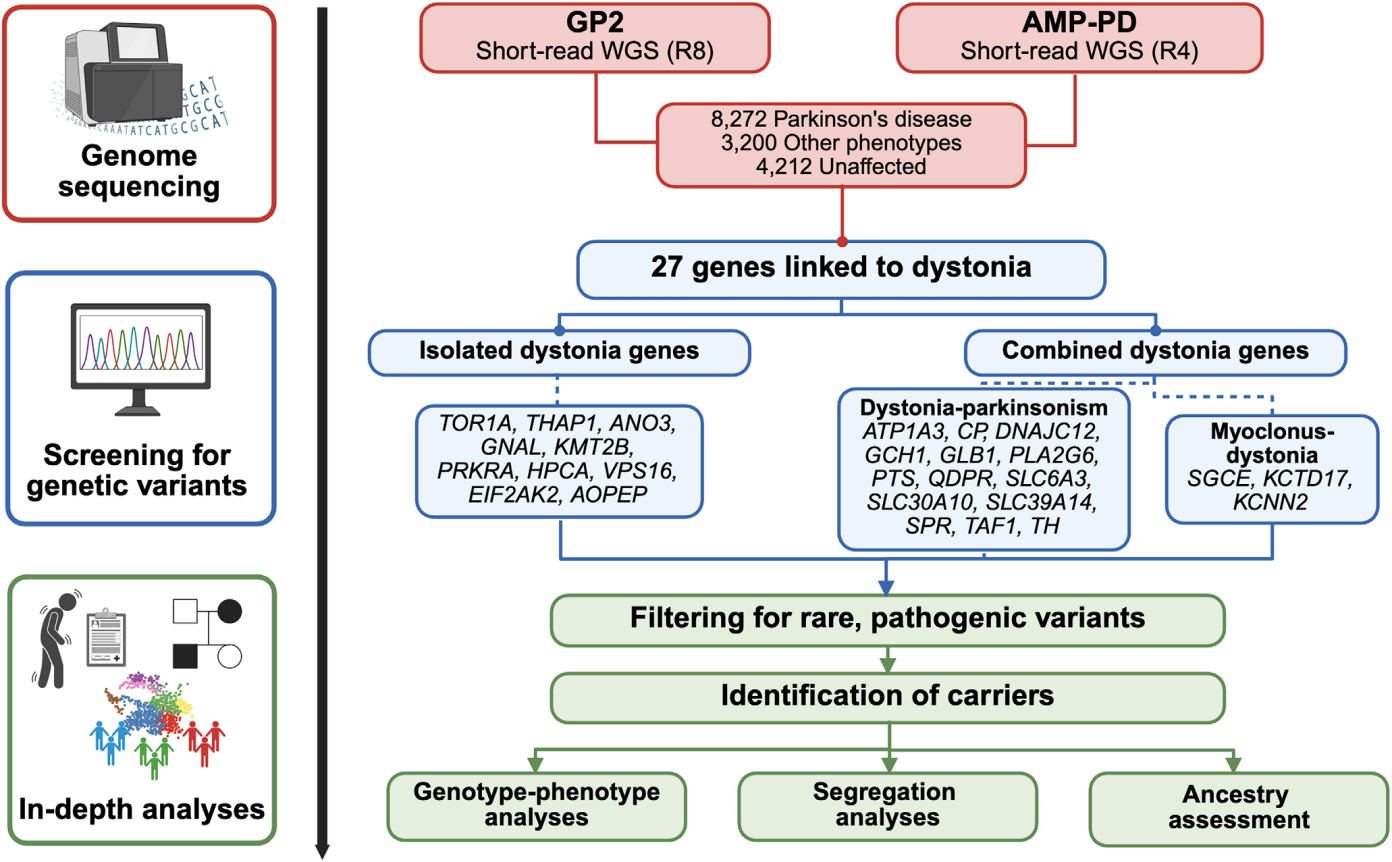
Study design and workflow. We screened short‐read whole‐genome sequencing (WGS) data from the Global Parkinson's Genetics Program (GP2, Release 8 [R8]) and the Accelerating Medicines Partnership‐Parkinson's Disease (AMP‐PD, Release 4 [R4]) for known variants in genes linked to different forms of dystonia. We included genes linked to isolated as well as combined dystonia phenotypes, the latter including dystonia‐parkinsonism and myoclonus‐dystonia. Only rare variants predicted to be pathogenic or likely pathogenic were included in further analyses. For identified carriers, we evaluated genotype–phenotype correlations, investigated segregation, and assessed ancestry distributions, where possible. This figure was created using BioRender. [Color figure can be viewed at wileyonlinelibrary.com]

Figure 1 shows our workflow. We analyzed short‐read sequencing data from 15,684 individuals of 11 genetically determined ancestries from GP2's Data Release 8 (DOI 10.5281/zenodo.13755496) and AMP‐PD's Release 4, including 8272 PD patients, 3200 individuals with atypical parkinsonism and other neurological/neurodegenerative phenotypes, and 4212 unaffected individuals (see Supplementary Material). The cohort characteristics are summarized in Table S1.

We investigated variants in genes linked to dystonia following the recommendations of the *MDS Task Force on the Nomenclature of Genetic Movement Disorders* (2): (1) isolated dystonia: *ANO3*, *AOPEP*, *EIF2AK2*, *GNAL*, *HPCA*, *KMT2B*, *PRKRA*, *THAP1*, *TOR1A*, and *VPS16*; (2) dystonia‐parkinsonism: *ATP1A3*, *CP*, *DNAJC12*, *GCH1*, *GLB1*, *PLA2G6*, *PTS*, *QDPR*, *SLC6A3*, *SLC30A10*, *SLC39A14*, *SPR*, *TAF1*, and *TH*; and (3) myoclonus‐dystonia: *SGCE*, *KCTD17*, and *KCNN2*. We filtered for rare (gnomAD minor allele frequency ≤1%) variants classified as pathogenic/likely pathogenic according to ClinVar (https://www.ncbi.nlm.nih.gov/clinvar/) and the American College of Medical Genetics and Genomics (ACMG) criteria^14^ (see Supplementary Material Fig. S1). For KMT2B, episignatures were determined to support pathogenicity evaluation (see Supplementary Material Table S4). For recessive genes, we screened for pathogenic/likely pathogenic homozygous variants or two pathogenic/likely pathogenic heterozygous variants; carriers of one pathogenic/likely pathogenic and one variant of uncertain significance were not considered as potentially compound heterozygous.

## Data and Code Availability

Data used in the preparation of this article were obtained from the GP2 (https://gp2.org). Specifically, we used tier 2 data from GP2 release 8 (DOI 10.5281/zenodo.13755496). Tier 1 data can be accessed by completing a form on the AMP‐PD website (https://amp-pd.org/register-for-amp-pd). Tier 2 data access requires approval and a Data Use Agreement signed by the institution. AMP‐PD data can be accessed through the AMP‐PD website (https://amp-pd.org). Qualified researchers are encouraged to apply for direct access to the data through AMP‐PD.

All codes generated for this article, and the identifiers for all software programs and packages used, are available on GitHub (https://github.com/GP2code/dystonia-genes-inPD) and were given a persistent identifier via Zenodo (DOI 10.5281/zenodo.15676002).

## Results

We identified 45 individuals from 6 ancestries carrying 26 distinct pathogenic/likely pathogenic variants in 9 dystonia‐linked genes. The identified pathogenic/likely pathogenic variants and their pathogenicity evaluation are summarized in Table 1. All 45 individuals were diagnosed with PD (n = 45/8272, 0.54%), whereas no carriers were diagnosed with atypical parkinsonism or other neurological/neurodegenerative diseases (n = 0/3200, 0%) or among unaffected individuals (n = 0/4212, 0%). The majority (n = 43) were carriers of heterozygous variants in genes linked to dominantly inherited dystonia genes (*ATP1A3*, *GCH1*, *SGCE*, *KMT2B*, *THAP1*, *TOR1A*, and *VPS16*), whereas only one carrier of a homozygous *PLA2G6* variant and one of compound‐heterozygous *PTS* variants (Fig. S3) were identified.

**TABLE 1:** Overview of identified pathogenic and likely pathogenic variants in dystonia‐linked genes

Gene	Chromosomal position	cDNA change	Protein change	Variant type	Zygosity	Pathogenicity	ACMG criteria^a^	Franklin	Varsome	MDSGene	ClinVar	CADD	GnomAD AF	n Carrier
Pathogenic variants in combined dystonia‐parkinsonism genes														
*ATP1A3*	chr19:41978053:T:C	c.1826A>G	p.Asp609Gly	Missense	Het	P/LP	PP3 (strong), PM2, PM5, PP2	LP	LP	NA	NA	32	NA	1
*GCH1*	chr14:54844023:C:G	c.747G>C	p.Arg249Ser	Missense	Het	P/LP	PM1, PM2, PM5, PP2, PP3	LP	VUS	Probably pathogenic	Uncertain significance	22.7	NA	1
	chr14:54844067:G:A	c.703C>T	p.Arg235Trp	Missense	Het	P/LP	PM1, PM2, PP2, PP3	LP	VUS	Probably pathogenic	NA	32	NA	1
	chr14:54844080:C:T	c.690G>A	p.Met230Ile	Missense	Het	P/LP	PM1, PM2, PP2, PP3	LP	LP	Possibly pathogenic	NA	26.2	NA	1
	chr14:54844099:T:C	c.671A>G	p.Lys224Arg	Missense	Het	P/LP	PM3 (strong), PM1, PM2, PP1, PP2, PP5	P	LB	Possibly pathogenic	Conflicting	21.3	0.000393846	10
	chr14:54844141:T:A	c.629A>T	p.His210Leu	Missense	Het	P/LP	PM1, PM2, PP2, PP3	LP	LP	NA	NA	27.5	NA	1
	chr14:54845766:AC:A	c.626 + 1del	NA	Splicing	Het	P/LP	PVS1, PM2	LP	LP	NA	NA	32	NA	1
	chr14:54845784:C:T	c.610G>A	p.Val204Ile	Missense	Het	P/LP	PS4, PM1, PM2, PP2, PP3, PP5	P	VUS	Possibly pathogenic	Conflicting	24.9	0.000124752	3
	chr14:54845787:C:T	c.607G>A	p.Gly203Arg	Missense	Het	P/LP	PP1 (strong), PS2, PS3 (supporting), PM1, PM2, PP2, PP3, PP5	P	P	Probably pathogenic	Pathogenic	29.1	NA	1
	chr14:54845837:G:C	c.557C>G	p.Thr186Arg	Missense	Het	P/LP	PM1, PM2, PM5, PP2, PP3	LP	P	NA	NA	28.8	NA	1
	chr14:54902414:C:A	c.250G>T	p.Glu84^a^	Nonsense	Het	P/LP	PVS1, PS4 (moderate), PM2, PP5	P	P	Probably pathogenic	Uncertain significance	41	NA	1
*PLA2G6*	chr22:38132917:C:A	c.991G>T	p.Asp331Tyr	Missense	Hom	P/LP	PM3 (strong), PS3 (supporting), PM1, PM2, PP2, PP3, PP5	P	VUS	NA	Pathogenic	26.0	0.0000262509	1
*PTS*	chr11:112233178:C:T	c.259C>T	p.Pro87Ser	Missense	Comphet	P/LP	PM3 (very strong), PS3, PM1, PM2, PM5, PP2, PP3, PP5	P	LP	Probably pathogenic	Pathogenic	23.3	0.000013138	1
	chr11:112233191:A:G	c.272A>G	p.Lys91Arg	Missense	Comphet	P/LP	PM3 (very strong), PM1, PM2, PM5, PP2, PP3, PP5	P	LP	Probably pathogenic	Pathogenic	22.8	NA	1
Pathogenic variants in myoclonus‐dystonia genes														
*SGCE*	chr7:94603289:C:G	c.825 + 1G>C	NA	Splicing	Het	P/LP	PVS1, PM2	LP	LP	Probably pathogenic	NA	33	NA	1
Pathogenic variants in isolated dystonia genes														
*KMT2B*	chr19:35728121:G:A	c.4521G>A	p.Trp1507^a^	Nonsense	Het	P/LP	PVS1, PM2	LP	LP	NA	NA	43	NA	1
*THAP1*	chr8:42839235:T:G	c.218A>C	p.Lys73Thr	Missense	Het	P/LP	PM1, PM2, PP2, PP3	LP	VUS	Possibly pathogenic	NA	25.2	0.00000656858	2
	chr8:42843044:G:C	c.51C>G	p.Asp17Glu	Missense	Het	P/LP	PM1, PM2, PM5, PP2	LP	VUS	Possibly pathogenic	NA	13.16	NA	1
	chr8:42843084:G:T	c.11C>A	p.Ser4Tyr	Missense	Het	P/LP	PM1, PM2, PM5, PP2, PP3	LP	LP	Possibly pathogenic	NA	25.7	NA	1
*TOR1A*	chr9:129814061:TCTC:T	c.907_909del	p.Glu303del	Inframe deletion	Het	P/LP	PM3 (very strong), PS3 (supporting), PM2, PM4, PP5	P	VUS	NA	Pathogenic/likely pathogenic	19.47	0.0000525348	2
	chr9:129814108:C:T	c.863G>A	p.Arg288Gln	Missense	Het	P/LP	PS4, PM2, PP5	LP	VUS	Probably pathogenic	Pathogenic/likely pathogenic	23.0	0.000059149	1
*VPS16*	chr20:2860067:C:A	c.156C>A	p.Asn52Lys	Missense	Het	P/LP	PS1, PM2, PP5, BP1	VUS	VUS	Probably pathogenic	Pathogenic	18.80	0.00007435	8
	chr20:2860458:C:T	c.379C>T	p.Gln127^a^	Nonsense	Het	P/LP	PVS1, PM2	LP	LP	NA	NA	37	NA	1
	chr20:2863064:G:T	c.1332‐1G>T	NA	Splicing	Het	P/LP	PVS1, PM2	LP	LP	NA	Uncertain significance	31	0.0000262902	1
	chr20:2864993:G:GT	c.1943dup	p.Ala649Serfs*16	Frameshift	Het	P/LP	PVS1, PM2	LP	LP	NA	NA	33	NA	1
	chr20:2866316:G:A	c.2375 + 1G>A	NA	Splicing	Het	P/LP	PVS1, PM2	LP	LP	NA	NA	34	NA	1

ACMG, American College of Medical Genetics and Genomics; CADD, Combined Annotation Dependent Depletion (https://cadd.gs.washington.edu/), ClinVar (https://www.ncbi.nlm.nih.gov/clinvar/), Franklin (https://franklin.genoox.com), GnomAD (https://gnomad.broadinstitute.org, version 4.1.0), MDSGene (https://www.mdsgene.org/), and Varsome (https://varsome.com/).

Abbreviations: cDNA, complementary DNA; het, heterozygous; AF, allele frequency; P/LP, pathogenic/likely pathogenic; NA, not available/not applicable; VUS, variant of uncertain significance; LB, likely benign; hom, homozygous; comphet, compound heterozygous.

^a^ According to Franklin and Varsome.

Overall, variants in *GCH1* were the most frequent (n = 21/45, 46.7%), followed by *VPS16* (n = 12/45, 26.7%). Particularly, there was one ancestry‐specific recurrent variant in both these genes: *GCH1* p.Lys224Arg, carried by 10 European‐ancestry individuals, including 2 siblings, and *VPS16* p.Asn52Lys, carried by 8 East Asian individuals. According to gnomAD, both variants are more frequent in these respective ancestral groups compared to other ancestries; however, the frequency in PD patients in our study was higher compared to ancestry‐matched controls (Table S2). With respect to additional variants in PD‐linked genes, 8 of the 45 individuals carried *GBA1* or *LRRK2* risk variants, and 5 carried single heterozygous pathogenic variants in *PRKN*, a recessively inherited PD gene (Table 2), but none harbored a disease‐explaining variant in an established PD‐linked gene (including *LRRK2*, *SNCA*, *VPS35*, *RAB32*, *PINK1*, *PRKN*, and *PARK7/DJ‐1*).

**TABLE 2:** Demographic and clinical characteristics of identified variant carriers

ID	Gender	Age	Genetic ancestry	Gene	Variant	Additional genetic finding	Diagnosis	AAO/AAD^a^	Symptom at onset	Levodopa responsive	FH PD	FH dystonia	Details FH	Signs of dystonia	Atypical signs
Pathogenic variants in combined dystonia‐parkinsonism genes															
GP2‐ID‐1	Male	33	EUR	*ATP1A3*	p.Asp609Gly		PD	29	NA	Yes	No	No	NA	Severe *off* dystonia	NA
GP2‐ID‐2	Male	82	EAS	*GCH1*	p.Arg249Ser		PD	67	Tremor and slowness	Yes	Yes	No	Brother with PD	No	No; dementia after 10 years disease duration
GP2‐ID‐3	Female	39	AAC	*GCH1*	p.Arg235Trp	*GBA1* rs3115534‐G	PD	35	Gait disorder	Yes	No	No	NA	Cervical dystonia	Mental retardation
GP2‐ID‐4	Female	47	CAS	*GCH1*	p.Met230Ile		PD	43	NA	NA	No	NA	NA	NA	NA
GP2‐ID‐5^b^	Female	77	EUR	*GCH1*	p.Lys224Arg		PD	71	NA	NA	Yes	NA	Sister with PD	NA	No
GP2‐ID‐6^b^	Female	74	EUR	*GCH1*	p.Lys224Arg		PD	59	NA	NA	Yes	NA	Sister with PD	NA	No
GP2‐ID‐7	Male	55	EUR	*GCH1*	p.Lys224Arg		PD	37	NA	NA	Yes	NA	Father with possible PD (not diagnosed)	NA	NA
AMPPD‐ID‐1	Female	53	EUR	*GCH1*	p.Lys224Arg		PD	NA	NA	NA	NA	NA	NA	NA	NA
AMPPD‐ID‐2	Female	62	EUR	*GCH1*	p.Lys224Arg		PD	62	NA	NA	Yes	NA	NA	NA	NA
GP2‐ID‐8	Male	68	EUR	*GCH1*	p.Lys224Arg		PD	68	Rigidity and bradykinesia	Yes	Yes	No	Father with PD	No	No
GP2‐ID‐9	Male	43	EUR	*GCH1*	p.Lys224Arg	*GBA1* p.Arg296Gln	PD	34	Aching shoulder, dragging left leg	Yes	Yes	No	Paternal uncle with PD	Slight *off* dystonia	No
AMPPD‐ID‐3	Female	60	EUR	*GCH1*	p.Lys224Arg		PD	NA	NA	NA	No	NA	NA	NA	NA
AMPPD‐ID‐4	Male	72	EUR	*GCH1*	p.Lys224Arg		PD	69	NA	NA	No	NA	NA	NA	NA
AMPPD‐ID‐5	Male	72	EUR	*GCH1*	p.Lys224Arg		PD	NA	NA	NA	NA	NA	NA	NA	NA
GP2‐ID‐10	Female	73	MDE	*GCH1*	p.His210Leu	PRKN deletion (het)	PD	56	Tremor	Yes	Yes	No	Brother with PD	No	No
AMPPD‐ID‐6	Male	56	EUR	*GCH1*	c.626 + 1del		PD	NA	NA	NA	NA	NA	NA	NA	NA
GP2‐ID‐11	Male	57		*GCH1*	p.Val204Ile		PD	54	Involuntary jaw and tongue movement, rigidity, dragging right foot	Yes	Yes	No	Father with PD	No	No
GP2‐ID‐12	Male	49	EUR	*GCH1*	p.Val204Ile		PD	43	Loss of dexterity	Yes	No	No	Not applicable	No	No
GP2‐ID‐13	Male	59	EUR	*GCH1*	p.Val204Ile		PD	38	Action tremor	Yes	No	No	NA	No	No
AMPPD‐ID‐7	Male	57	EUR	*GCH1*	p.Gly203Arg		PD	57	NA	NA	No	NA	NA	NA	NA
GP2‐ID‐14	Female	47	EUR	*GCH1*	p.Thr186Arg		PD	32	NA	NA	No	NA	NA	NA	NA
AMPPD‐ID‐8	Male	73	EUR	*GCH1*	p.Glu84^b^		PD	NA	NA	NA	NA	NA	NA	NA	NA
GP2‐ID‐15	Female	40	EAS	*PLA2G6*	p.Asp331Tyr	*LRRK2* p.Gly2385Arg	PD	27	Right‐leg slowness	Yes	No	Yes	Consanguineous family	Feet and hand dystonia	Severe depression
GP2‐ID‐16	Male	48	EAS	*PTS*	p.Pro87Ser; p.Lys91Arg		PD	43	Right‐leg dystonia	Yes	NA	NA	NA	Right‐leg dystonia while walking	No
Pathogenic variants in myoclonus‐dystonia genes															
GP2‐ID‐19	Female	47	EUR	*SGCE*	c.825 + 1G>C		PD	40	NA	NA	No	NA	NA	NA	NA
Pathogenic variants in isolated dystonia genes															
AMPPD‐ID‐14	Female	58	AJ	*KMT2B*	p.Trp1507^b^		PD	58	NA	NA	NA	NA	NA	NA	NA
GP2‐ID‐20^b^	Female	46	EUR	*THAP1*	p.Lys73Thr		PD	38	Tremor, rigidity	Yes	Yes	No	Mother with PD	Slight *off* dystonia	No
GP2‐ID‐21^b^	Female	71	EUR	*THAP1*	p.Lys73Thr	*PRKN* p.Pro132Thrfs × 9 (het)	PD	67	Foot cramps	Yes	Yes	No	Daughter with PD	Slight *off* dystonia	No
GP2‐ID‐22	Male	59	EUR	*THAP1*	p.Asp17Glu		PD	38	NA	NA	Yes	NA	Mother with parkinsonism	NA	No
GP2‐ID‐23	Female	50	EAS	*THAP1*	p.Ser4Tyr	PRKN deletion (het)	PD	40	Gait disturbance	Yes	Yes	No	Brother with PD	No	No
AMPPD‐ID‐15	Female	55	EUR	*TOR1A*	p.Glu303del		PD	55	NA	NA	No	NA	NA	NA	NA
GP2‐ID‐24	Male	NA	AJ	*TOR1A*	p.Glu303del		PD	72	Spatial difficulties	Yes	Yes	Yes	Mother with PD; daughter and two granddaughters with dystonia	No	Memory loss, visual hallucination, RBD
GP2‐ID‐25	Female	62	EUR	*TOR1A*	p.Arg288Gln	*GBA1* p.Asn409Ser	PD	44	Tremor, stiffness in the neck	Yes	Possibly	No	Maternal grandfather with possible PD; mother with AD	Mild dystonic posture in left arm	No
GP2‐ID‐26	Male	67	EAS	*VPS16*	p.Asn52Lys		PD	48	Upper‐limb tremors, bradykinesia	Yes	No	No	Not applicable	Right striatal toe, mild camptocormia, and Pisa syndrome	No
GP2‐ID‐27	Male	53	EAS	*VPS16*	p.Asn52Lys		PD	33	Tremor, stiffness, slow movements	Yes^c^	No	No	Not applicable	Blepharospasm	No
GP2‐ID‐28	Male	49	EAS	*VPS16*	p.Asn52Lys	*GBA1* p.Leu483Arg, *PINK1* p.Leu347Pro (het)	PD	47	Chest muscle pain, limb tremor	Yes^c^	No	No	Not applicable	Flexed posture of the left hand, intermittent slight *off* dystonia	No
GP2‐ID‐29	Female	51	EAS	*VPS16*	p.Asn52Lys	PRKN deletion (het)	PD	47	Tremor	Yes	No	No	Not applicable	No	No
GP2‐ID‐30	Female	48	EAS	*VPS16*	p.Asn52Lys	*GBA1* p.Arg502Cys	PD	48	Right‐hand tremor	No	Yes	No	Father with PD	No	No
GP2‐ID‐31	Male	68	EAS	*VPS16*	p.Asn52Lys		PD	58	Unk	Yes^c^	Yes	No	Mother and brother with PD	No	No
GP2‐ID‐32	Female	51	EAS	*VPS16*	p.Asn52Lys	*LRRK2* p.Arg1628Pro	PD	34	Body weakness associated with cramps	Yes	No	No	Not applicable	Cervical dystonia	Postural hypotension, frequent falls
GP2‐ID‐33	Male	70	EAS	*VPS16*	p.Asn52Lys		PD	43	Rest tremor, micrographia, slowness in initiating movement	Yes	No	No	Not applicable	No	No
GP2‐ID‐34	Male	78	EUR	*VPS16*	p.Gln127^b^		PD	69	NA	NA	Not Reported	NA	NA	NA	Pathology diagnoses: LBD, CAA, AD
GP2‐ID‐35	Female	45	EUR	*VPS16*	c.1332‐1G>T	*GBA1* RecNciI	PD	36	Slowness	Yes	No	No	NA	Limb dystonia (with *on* exaggeration)	No
GP2‐ID‐37	Female	59	EAS	*VPS16*	p.Ala649Serfs × 16		PD	59	Tremor	Yes^c^	Yes	No	Paternal aunt with PD; multiple family members (eg, father and brother) with tremor	Leg dystonia (probably in *off*‐medication state)	No
GP2‐ID‐38	Male	93	EUR	*VPS16*	c.2375 + 1G>A		PD	83	NA	NA	Not reported	NA	NA	NA	Pathology diagnoses: LBD, ARTAG, FTLD, SVD

Additional genetic findings: *LRRK2* p.R1628P and p.G2385R are considered risk factor variants for PD; for example, carriers have an increased risk of developing PD, but these variants are not considered causal (“monogenic”). Similarly, in the context of PD, all variants in *GBA1* are considered risk factors but not causal.

Abbreviations: AAO, age at onset; AAD, age at diagnosis; FH, family history; PD, Parkinson's disease; GP2, Global Parkinson's Genetics Program; EUR, European; MDE, Middle Eastern ancestry; NA, not available/not applicable; AJ, Ashkenazi Jewish; EAS, East Asian ancestry; AAC, African admixed ancestry; CAS, Central Asian ancestry; het, heterozygous; RBD, rapid eye movement sleep behavior disorder; AD, Alzheimer's disease; LBD, Lewy body dementia; CAA, cerebral amyloid angiopathy; ARTAG, aging‐related tau astrogliopathy; FTLD, frontotemporal lobar degeneration; SVD, small vessel disease; Unk, Unknown.

^a^ When both were available, the AAO is presented. For those individuals without an available AAO, we indicate the AAD.

^b^ Related individuals within the investigated cohort. GP2‐ID‐5 and GP2‐ID‐6 are siblings, and GP2‐ID‐20 is the daughter of GP2‐ID‐21.

^c^ Individuals who underwent deep brain stimulation.

Table 2 presents the demographic and clinical characteristics of identified variant carriers. The majority with available data on treatment exhibited a good response to levodopa (n = 25/26, missing for n = 19). The median age at PD motor symptom onset (age at onset [AAO]) or PD diagnosis (AAD) across all carriers was 47 years (interquartile range: 38–59 years), ranging from 27 to 83 years. Fourteen carriers exhibited variable signs of dystonia (information unavailable for n = 19), which was believed to be attributed to motor fluctuations due to dopaminergic medications (*off* dystonia) in a subset. Whereas 17 (42.5%, unknown for n = 5) carriers had a positive family history of PD, only 2 individuals had family members with dystonia (unknown for n = 19).

Interestingly, 1 late‐onset PD patient (AAO 72 years) without dystonia, carrying the pathogenic *TOR1A* p.Glu303del variant, had a mother with PD, but also 1 daughter and 2 grandchildren with dystonia (Fig. S2A). Unfortunately, no additional family members were available for genetic testing.

We also identified a pathogenic *THAP1* variant (p.Lys73Thr) in an individual and her mother (Fig. S2B), both diagnosed with PD and slight foot *off* dystonia. Interestingly, the mother's first motor symptom (AAO 67 years) were foot cramps, whereas her daughter exhibited no additional signs of possible dystonia and had a younger AAO of 38 years.

In addition to these 45 individuals, we identified 10 carriers of single heterozygous pathogenic variants in recessively inherited dystonia genes, including *AOPEP* (n = 5), *SPR* (n = 4), and *HPCA* (n = 1) (Table S3). We did not detect any pathogenic/likely pathogenic variants in *ANO3*, *EIF2AK2*, *GNAL*, *PRKRA*, *CP*, *DNAJC12*, *GLB1*, *QDPR*, *SLC6A3*, *SLC30A10*, *SLC39A14*, *TAF1*, *TH*, *KCTD17*, and *KCNN2*. However, 613 individuals carried rare variants of uncertain significance across all investigated genes (580 with PD, 8 with atypical parkinsonism, and 25 unaffected).

## Discussion

Pathogenic variants in dystonia‐linked genes were found in less than 1% of PD patients. Despite being rare, they were enriched in PD patients compared to controls and individuals with atypical parkinsonism, where no such variants were identified.

Not surprisingly and in line with previous studies,^10^, ^11^ variants in *GCH1* were relatively frequent. *GCH1* is known to cause dystonia and parkinsonism, independently or combined. According to a systematic review, 86% of heterozygous *GCH1* carriers present with dystonia to a variable extent, either isolated or combined dystonia‐parkinsonism, whereas 11% have only parkinsonism.^15^ Several case series reported PD patients harboring pathogenic *GCH1* with abnormal DaTscan imaging, indicating nigrostriatal dopaminergic deficits consistent with neurodegenerative parkinsonism.^11^, ^16^, ^17^ Another study suggested *GCH1* variants may lead to parkinsonism by unmasking subclinical nigral pathology, not by causing nigral neurodegeneration.^18^ Additionally, according to the most recent PD genome‐wide association studies (GWAS), common variants were identified as the likely risk factor in the *GCH1* locus.^19^, ^20^ Particularly, we identified one recurrent *GCH1* variant (p.Lys224Arg) present only in European‐ancestry individuals. Although this variant has a conflicting ClinVar interpretation, it was found only in PD patients and absent from controls in our study, and it was significantly more frequent in European‐ancestry PD patients from our study compared to ancestry‐matched controls obtained from gnomAD (Table S2). Further, it was present in 2 PD patients from the same family (sisters), further supporting its pathogenic role. In addition to *GCH1*, variants in genes linked to dystonia‐parkinsonism (ie, *ATP1A3*, *PLA2G6*, and *PTS*) collectively accounted for more than half of the identified carriers overall (24/45, 53%).

Interestingly, 47% (n = 21/45) of all identified carriers harbored variants in isolated dystonia genes, where parkinsonism is unexpected.^21^, ^22^ Among those genes was *VPS16*, with the second‐largest number of pathogenic variants (n = 9) observed in this study. We identified one recurrent *VPS16* variant, exclusively present in East Asian individuals. Although it was predicted to be pathogenic on ClinVar and reported as disease‐causing in Chinese dystonia patients in the homozygous state,^23^ the overall pathogenicity evaluation remains partially conflicting because this variant is relatively frequent in East Asians controls. In our study, the frequency in East Asian PD patients was higher compared to ancestry‐matched controls obtained from gnomAD, though this trend was not statistically significant (Table S2). Overall, whether these findings in isolated dystonia genes indicate a causal relationship and contribute to the parkinsonian phenotype (variable clinical expressivity) remains elusive. Although, interestingly, pure parkinsonism has recently been suggested as a potential phenotype expansion for *THAP1*‐related disorders,^24^ another, probably more likely, explanation might be that these are incidental findings reflecting reduced penetrance (for dystonia), a phenomenon well known in several monogenic forms of dystonia, especially in DYT‐*THAP1* (penetrance ~50%) and DYT‐*TOR1A* (penetrance ~30%).

Finally, we identified 1 carrier of a pathogenic variant in the myoclonus‐dystonia gene *SGCE*. Particularly, due to maternal imprinting, *SGCE* variants exhibit significantly reduced penetrance when inherited maternally, and the phenotype develops only if the variant is inherited paternally. Unfortunately, no additional family members were available for genetic testing, and we were unable to further investigate the inheritance pattern.

It is important to note that variant interpretation in the context of clinically and genetically heterogeneous movement disorders, particularly those with reduced or age‐dependent penetrance and variable expressivity, can be considerably more complex than for fully penetrant, single‐gene syndromes. However, in dystonia, the same variant may lead to a wide spectrum of clinical presentations—or no manifestation at all—making it difficult to assess pathogenicity based on genotype alone applying the ACMG criteria. This complexity underscores the need for cautious interpretation, especially when evaluating variants in genes not classically associated with the observed phenotype or when segregation and functional data are lacking. Another important complicating factor is the possibility of overlapping or interacting genetic contributions, further challenging interpretation. Among the 45 individuals carrying dystonia‐linked variants, 12 also harbored either PD‐associated risk variants or single heterozygous variants in recessive PD‐linked genes. Particularly, the latter does not exclude the possibility of an undetected second pathogenic allele. Separately, the concept of a potential combined effect from variants in different genes (“dual carriers”) is intriguing and has been hypothesized for PD in various contexts, though it has not been systematically investigated in large cohorts. Although we currently interpret these observations as incidental, future studies in expanded datasets will be important to formally assess these possibilities.

This study has some limitations. The availability of clinical data for samples obtained from AMP‐PD was very limited; detailed data on dystonia were not available, thereby not allowing us to perform meaningful genotype–phenotype analyses. Further, family members of identified variant carriers were unavailable for genetic testing within this study, precluding us from performing segregation analyses, which would be helpful in assessing pathogenicity, especially in case of genes with reduced penetrance.

In conclusion, pathogenic variants in dystonia‐linked genes were present, albeit rare, among PD patients and absent in individuals with atypical parkinsonism and controls. Although misdiagnosis cannot be excluded, available clinical and pathological data support a neurodegenerative disease in most cases. The enrichment in PD patients compared to controls suggests that dystonia gene variants may predispose to PD and that there may be potential biological overlap and shared pathways between dystonia and PD. Importantly, our results, alongside previous evidence from screening studies and GWAS, reinforce *GCH1* as a PD‐relevant gene holding clinical implications. Similarly, other genes associated with dystonia‐ parkinsonism may have clinical relevance for PD patients, whereas variants in isolated or myoclonus‐dystonia genes more likely represent incidental findings, potentially due to reduced penetrance. Collectively, our results highlight the need for careful interpretation and counseling in clinical genetics regarding a possible role of pathogenic dystonia gene variants in PD patients.

## Author Roles

(1) Research Project: A. Conception, B. Organization, C. Execution; (2) Statistical Analysis: A. Design, B. Execution, C. Review and Critique; (3) Manuscript Preparation: A. Writing of the First Draft, B. Review and Critique.

L.M.L.: 1A, 1B, 1C, 2A, 2B, 3A, 3B.

Z.H.F.: 1C, 2B, 2C.

A.H.T.: 1C, 3B.

R.N.A.: 1C, 3B.

R.A.: 1C, 3B.

R.B.: 1C, 3B.

M.F.: 1C, 3B.

J.L.I.F.: 1C, 3B.

S.J.: 1C, 3B.

Z.J.: 1C, 3B.

B.J.: 1C, 3B.

I.J.K.S.: 1C, 3B.

R.K.: 1C, 3B.

G.K.: 1C, 3B.

C.H.L.: 1C, 3B.

M.T.P.: 1C, 3B.

S.B.S.: 1C, 3B.

T.S.: 1C, 3B.

J.H.S.: 1C, 3B.

J.M.S.: 1C, 3B.

Y.W.T.: 1C, 3B.

R.U.: 1C, 3B.

T.W.: 1C, 3B.

Z.K.W.: 1C, 3B.

L.W.: 1C, 3B.

R.M.W.: 1C, 3B.

K.E.Z.: 1C, 3B.

C.B.: 1C, 2A, 2C, 3B.

A.S.: 1C, 2A, 2C, 3B.

H.R.M.: 1C, 2A, 2C, 3B.

N.E.M.: 1C, 2A, 2C, 3B.

S.H.L.: 1C, 3B.

K.L.: 1A, 1B, 2A, 2C, 3B.

C.K.: 1A, 1B, 2A, 3B.

## Full financial disclosures of all authors for the preceding 12 months

**L.M.L.:** stock ownership in medically related fields, none. Intellectual property rights, none. Consultancies, none. Expert testimony, none. Advisory boards, none. Employment, Laboratories of Neurogenetics, National Institute on Aging, Bethesda, MD, USA; University Hospital Schleswig Holstein, Campus Luebeck, Luebeck, Germany. Partnerships, none. Inventions, none. Contracts, none. Honoraria, MDS Faculty Honoraria (July 2024 and March 2025). Royalties, none. Patents, none. Grants, none. Other, Bachmann Strauss Dystonia Fellowship Stipend (October 2021 to October 2024). **Z.‐H.F.:** stock ownership in medically related fields, none. Intellectual property rights, none. Consultancies, none. Expert testimony, none. Advisory boards, none. Employment, none. Partnerships, none. Inventions, none. Contracts, contract with The Michael J. Fox Foundation for work on the Global Parkinson's Genetics Program. Honoraria, none. Royalties, none. Patents, None. Grants, none. Other, Aligning Science Across Parkinson's Initiative (Global Parkinson's Genetics Program). **L.S.:** stock ownership in medically related fields, none. Intellectual property rights, none. Consultancies, none. Expert testimony, none. Advisory boards, none. Employment, none. Partnerships, none. Inventions, none. Contracts, none. Honoraria, none. Royalties, none. Patents, none. Grants, none. Other, none. **A.H.T.:** stock ownership in medically related fields, none. Intellectual property rights, none. Consultancies, from Elsevier as section editor for *Parkinsonism and Related Disorders*. Expert testimony, none. Advisory boards, none. Employment, employed by Universiti Malaya. Partnerships, none. Inventions, none. Contracts, none. Honoraria, speakers’ honoraria from International Parkinson and Movement Disorders, Eisai, and Orion Pharma. Royalties, none. Patents, none. Grants, The Michael J. Fox Foundation and Global Parkinson's Genetics Program. Other, none. **R.N.A.:** stock ownership in medically related fields, none. Intellectual property rights, none. Consultancies, Biogen, Biohaven, Capsida, Gain Therapeutics, Genzyme/Sanofi, Janssen, Servier, SK Biopharmaceuticals, Takeda, and Vanqua Bio. Expert testimony, none. Advisory boards, none. Employment, none. Partnerships, none. Inventions, none. Contracts, none. Honoraria, none. Royalties, none. Patents, none. Grants, The Michael J. Fox Foundation, the Silverstein Foundation, and the Parkinson's Foundation. Other, none. **R.A.:** stock ownership in medically related fields, none. Intellectual property rights, none. Consultancies, none. Expert testimony, none. Advisory boards, none. Employment, Institut National Mongi Ben Hamida de Neurologie. Partnerships, none. Inventions, none. Contracts, none. Honoraria, none. Royalties, none. Patents, MJFF‐026144. Grants, none. Other, none. **R.B.:** stock ownership in medically related fields, none. Intellectual property rights, none. Consultancies, none. Expert testimony, none. Advisory boards, none. Employment, University of Tor Vergata. Partnerships, none. Inventions, none. Contracts, none. Honoraria, none. Royalties, none. Patents, none. Grants, PF‐VSA‐1300309 by Parkinson's Foundation. Other, none. **M.F.:** stock ownership in medically related fields, none. Intellectual property rights, none. Consultancies, none. Expert testimony, none. Advisory boards, none. Employment, Royal Free London NHS Foundation Trust, honorary contract University College London. Partnerships, none. Inventions, none. Contracts, none. Honoraria, none. Royalties, none. Patents, none. Grants, none. Other, none. **J.L.I.F.:** stock ownership in medically related fields, none. Intellectual property rights, none. Consultancies, none. Expert testimony, none. Advisory boards, none. Employment, Royal Free London NHS Foundation Trust. Partnerships, none. Inventions, none. Contracts, none. Honoraria, none. Royalties, none. Patents, none. Grants, none. Other, none. **J.J.:** stock ownership in medically related fields, none. Intellectual property rights, none. Consultancies, none. Expert testimony, none. Advisory boards, none. Employment, none. Partnerships, none. Inventions, none. Contracts, none. Honoraria, none. Royalties, none. Patents, none. Grants, none. Other, none. **S.J.:** stock ownership in medically related fields, none. Intellectual property rights, none. Consultancies, none. Expert testimony, none. Advisory boards, none. Employment, University College London. Partnerships, none. Inventions, none. Contracts, none. Honoraria, none. Royalties, none. Patents, none. Grants, none. Other, none. **Z.J.:** stock ownership in medically related fields, none. Intellectual property rights, none. Consultancies, none. Expert testimony, none. Advisory boards, none. Employment, University College London. Partnerships, none. Inventions, none. Contracts, none. Honoraria, none. Royalties, none. Patents, none. Grants, MRCMR/Y00440X/1, RLW BRAIN grant, and MJFF. Other, none. **B.J.:** stock ownership in medically related fields, none. Intellectual property rights, none. Consultancies, Hanwha Pharma and SK Biopharm. Expert testimony, none. Advisory boards, none. Employment, none. Partnerships, none. Inventions, none. Contracts, none. Honoraria, none. Royalties, none. Patents, none. Grants, Zemvax and Kael. Other, none. **I.J.K.S**.: stock ownership in medically related fields, none. Intellectual property rights, none. Consultancies, none. Expert testimony, none. Advisory boards, none. Employment, Northwestern University. Partnerships, none. Inventions, none. Contracts, none. Honoraria, none. Royalties, none. Patents, none. Grants, MJFF‐023355. Other, none. **R.K**.: stock ownership in medically related fields, none. Intellectual property rights, none. Consultancies, Bial and AbbVie. Expert testimony, none. Advisory boards, AbbVie and Roche. Employment, LIH, LCSB, and CHL. Partnerships, one. Inventions, none. Contracts, none. Honoraria, none. Royalties, none. Patents, none. Grants, FNR, MJFF, and EU‐Horizon. Other, none. **G.K.:** stock ownership in medically related fields, none. Intellectual property rights, none. Consultancies, none. Expert testimony, none. Advisory boards, none. Employment, Department of Neurology, Kiel University. Partnerships, none. Inventions, none. Contracts, none. Honoraria, none. Royalties, none. Patents, none. Grants, BMBF (German Ministry of Education and Research) grant for the investigation of the Genetics of Antibody Associated Encephalitis. Other, none. **C.‐H.L**.: stock ownership in medically related fields, none. Intellectual property rights, none. Consultancies, none. Expert testimony, none. Advisory boards, none. Employment, Department of Neurology, National Taiwan University Hospital, Taipei, Taiwan; and Department of Biomedical Engineering, National Taiwan University, Taipei, Taiwan. Partnerships, none. Inventions, none. Contracts, none. Honoraria, speakers' honoraria from Eisai. Royalties, none. Patents, none. Grants, National Science and Technology Council (NSTC) and National Health Research Institutes (NHRI). Other, none. **L.P.:** stock ownership in medically related fields, none. Intellectual property rights, none. Consultancies, none. Expert testimony, none. Advisory boards, none. Employment, none. Partnerships, none. Inventions, none. Contracts, none. Honoraria, none. Royalties, none. Patents, none. Grants, Thiemann Stiftung/Thiemann Fellowship prize 2024. Other, none. **M.T.P.:** stock ownership in medically related fields, none. Intellectual property rights, none. Consultancies, uMedeor. Expert testimony, none. Advisory boards, none. Employment, FISEVI, Seville, Spain. Partnerships, none. Inventions, none. Contracts, none. Honoraria, none. Royalties, none. Patents, none. Grants, Parkinson's UK. Other, none. **S.B.S**.: stock ownership in medically related fields, none. Intellectual property rights, none. Consultancies, none. Expert testimony, none. Advisory boards, none. Employment, none. Partnerships, none. Inventions, none. Contracts, none. Honoraria, MDS Faculty Honoraria. Royalties, none. Patents, none. Grants, MJFF‐026144. Other, none. **T.S**.: stock ownership in medically related fields, none. Intellectual property rights, none. Consultancies, none. Expert testimony, none. Advisory boards, none. Employment, none. Partnerships, none. Inventions, none. Contracts, none. Honoraria, none. Royalties, none. Patents, none. Grants, PRIN2022 by the Italian Ministry of University. Other, none. **J.H.S.:** stock ownership in medically related fields, none. Intellectual property rights, none. Consultancies, SK Chemical and Esai Korea. Expert testimony, none. Advisory boards, none. Employment, none. Partnerships, none. Inventions, none. Contracts, none. Honoraria, MDS faculty and KMDS faculty honoraria. Royalties, none. Patents, none. Grants, National Research Foundation (NRF) grants funded by the Ministry of Science and (ICT) and by the Ministry of Education in South Korea. Other, none. **J.M.S**.: stock ownership in medically related fields, none. Intellectual property rights, none. Consultancies, none. Expert testimony, none. Advisory boards, Helis Medical Research Foundation. Employment, Baylor College of Medicine. Partnerships, one. Inventions, none. Contracts, none. Honoraria, none. Royalties, none. Patents, none. Grants, US National Institutes of Health and Silverstein Foundation for GBA1 Parkinson's Disease. Other, none. **Y.W.T**.: stock ownership in medically related fields, none. Intellectual property rights, none. Consultancies, none. Expert testimony, none. Advisory boards, none. Employment, none. Partnerships, none. Inventions, none. Contracts, none. Honoraria, none. Royalties, none. Patents, none. Grants, none. Other, none. **R.U.:** stock ownership in medically related fields, none. Intellectual property rights, none. Consultancies, none. Expert testimony, none. Advisory boards, none. Employment, Mayo Clinic, Jacksonville, FL, USA. Partnerships, none. Inventions, none. Contracts, none. Honoraria, none. Royalties, none. Patents, none. Grants, none. Other, none. **T.W.:** stock ownership in medically related fields, none. Intellectual property rights, none. Consultancies, none. Expert testimony, none. Advisory boards, none. Employment, UCL, Queen Square Institute of Neurology. Partnerships, none. Inventions, none. Contracts, none. Honoraria, none. Royalties, none. Patents, none. Grants, MRCMR/Y00440X/1 and RLW BRAIN grant. Other, none. **Z.K.W**.: stock ownership in medically related fields, none. Intellectual property rights, none. Consultancies, Eli Lilly & Company and for Savanna; Biotherapeutics, Inc. Expert testimony, none. Advisory boards, Vigil Neuroscience, Inc. Employment, Mayo Clinic, Jacksonville, FL, USA. Partnerships, none. Inventions, none. Contracts, none. Honoraria, none. Royalties, none. Patents, none. Grants, none. Other, support from NIH/NIA and NIH/NINDS (1U19AG063911, FAIN: U19AG063911), Haworth Family Professorship in Neurodegenerative Diseases fund, the Albertson Parkinson's Research Foundation, PPND Family Foundation, and Margaret N. and John Wilchek Family. PI/Co‐PI on Biohaven Pharmaceuticals, Inc. (BHV4157‐206), Vigil Neuroscience, Inc., ONO‐2808‐03, and Amylyx AMX0035‐009. **L.W.:** stock ownership in medically related fields, none. Intellectual property rights, none. Consultancies, none. Expert testimony, none. Advisory boards, none. Employment, University College London Queen Square Institute of Neurology. Partnerships, none. Inventions, none. Contracts, none. Honoraria, none. Royalties, none. Patents, none. Grants, none. Other, none. **R.‐M.W**.: stock ownership in medically related fields, none. Intellectual property rights, none. Consultancies, none. Expert testimony, none. Advisory boards, none. Employment, National Taiwan University Hospital, College of Medicine, National Taiwan University. Partnerships, none. Inventions, none. Contracts, none. Honoraria, none. Royalties, none. Patents, none. Grants, National Science and Technology Council, National Taiwan University Hospital. Other, none. **K.E.Z.:** stock ownership in medically related fields, none. Intellectual property rights, none. Consultancies, consultant and received fees from Merz, Ipsen, Alexion, Bial, and the German Federal Institute for Drugs and Medical Devices (BfArM). Expert testimony, none. Advisory boards, none. Employment, Department of Neurology, Kiel University. Partnerships, none. Inventions, none. Contracts, none. Honoraria, Bayer Vital GmbH, Bial, AbbVie, Alexion, Allergan, and Merz outside the submitted work. Royalties, none. Patents, none. Grants, Research support from Strathmann and the German Research Council. Other, none. **C.B**.: stock ownership in medically related fields, none. Intellectual property rights, none. Consultancies, none. Expert testimony, none. Advisory boards, none. Employment, Laboratories of Neurogenetics, National Institute on Aging, Bethesda, MD, USA, and Coalition for Aligning Science. Partnerships, none. Inventions, none. Contracts, none. Honoraria, none. Royalties, none. Patents, none. Grants, none. Other, none. **A.S**.: stock ownership in medically related fields, spouse owns employment incentive‐related stock in GeneDx, a diagnostics company. Intellectual property rights, named as an inventor on patents for a diagnostic for stroke and for molecular testing for C9orf72 repeats. Consultancies, none. Expert testimony, none. Advisory boards, scientific advisory board of the Lewy Body Disease Association and for Cajal Neuroscience (both positions unpaid). Employment, none. Partnerships, none. Inventions, none. Contracts, none. Honoraria, honorarium for speaking at the World Laureates Association. Royalties, none. Patents, named as an inventor on patents for a diagnostic for stroke and for molecular testing for C9orf72 repeats. Grants, lead investigator for a grant from The Michael J. Fox Foundation for Parkinson's Research. Other, none. **N.E.M**.: stock ownership in medically related fields, none. Intellectual property rights, none. Consultancies, none. Expert testimony, none. Advisory boards, none. Employment, Northwestern University. Partnerships, none. Inventions, none. Contracts, none. Honoraria, honoraria from Parkinson's Foundation to be part of PDGENEration steering committee. Royalties, none. Patents, none. Grants, NIH NINDS K08 (1K08NS131581‐01A1). Other, none. **H.R.M**.: stock ownership in medically related fields, none. Intellectual property rights, none. Consultancies, Aprinoia. Expert testimony, none. Advisory boards, none. Employment, UCL. Partnerships, none. Inventions, none. Contracts, none. Honoraria, Movement Disorders Society. Royalties, none. Patents, co‐applicant on a patent application related to C9orf72—Method for Diagnosing a Neurodegenerative Disease (PCT/GB2012/052140). Grants, Parkinson's UK, Cure Parkinson's Trust, PSP Association, Medical Research Council, and The Michael J. Fox Foundation. Other, none. **S.‐Y.L**.: stock ownership in medically related fields, none. Intellectual property rights, none. Consultancies, consultancies from The Michael J. Fox Foundation for Parkinson's Research (MJFF) and the Aligning Science Across Parkinson's‐Global Parkinson's Genetics Program (ASAP‐GP2). Expert testimony, none. Advisory boards, honoraria for participating as a member of the Neurotorium Editorial Board. Employment, University of Malaya, Kuala Lumpur, Malaysia. Partnerships, none. Inventions, none. Contracts, none. Honoraria, honoraria for lecturing/teaching from the International Parkinson and Movement Disorder Society (MDS) and Medtronic; stipends from the MDS as chair of the Asian‐Oceanian Section, and *npj PD* as associate editor. Royalties, none. Patents, none. Grants, research grants from The Michael J. Fox Foundation (MJFF). Other, none. **K.L**.: stock ownership in medically related fields, none. Intellectual property rights, none. Consultancies, none. Expert testimony, none. Advisory boards, none. Employment, University of Luebeck, Luebeck, Germany. Partnerships, none. Inventions, none. Contracts, none. Honoraria, none. Royalties, none. Patents, none. Grants, Dystonia Medical Research Foundation and German Research Foundation (DFG). Other, none. **C.K.:** stock ownership in medically related fields, none. Intellectual property rights, none. Consultancies, medical advisor to Centogene and Biogen. Expert testimony, none. Advisory boards, none. Employment, University of Luebeck, Luebeck, Germany. Partnerships, none. Inventions, none. Contracts, none. Honoraria, speakers' honoraria from Bial. Royalties, Oxford University Press and Springer Nature. Patents, none. Grants, The Michael J. Fox Foundation for Parkinson's Research, Aligning Science Across Parkinson's Initiative, and German Research Foundation. Other, none.

## Supporting information


**Data S1.** Supporting Information.

## Data Availability

The data that support the findings of this study are openly available in GP2 Data Release 8 at https://gp2.org/the-components-of-gp2s-8th-data-release/, reference number DOI: 10.5281/zenodo.13755496.
